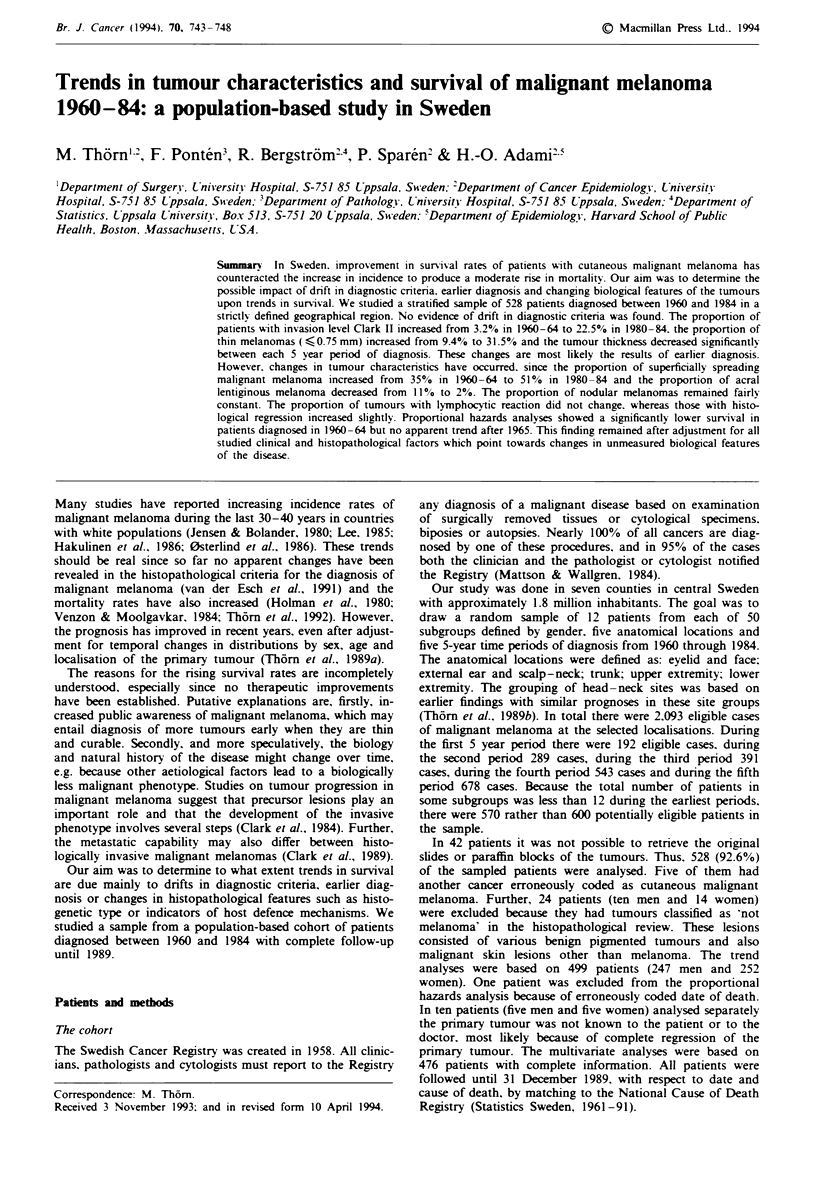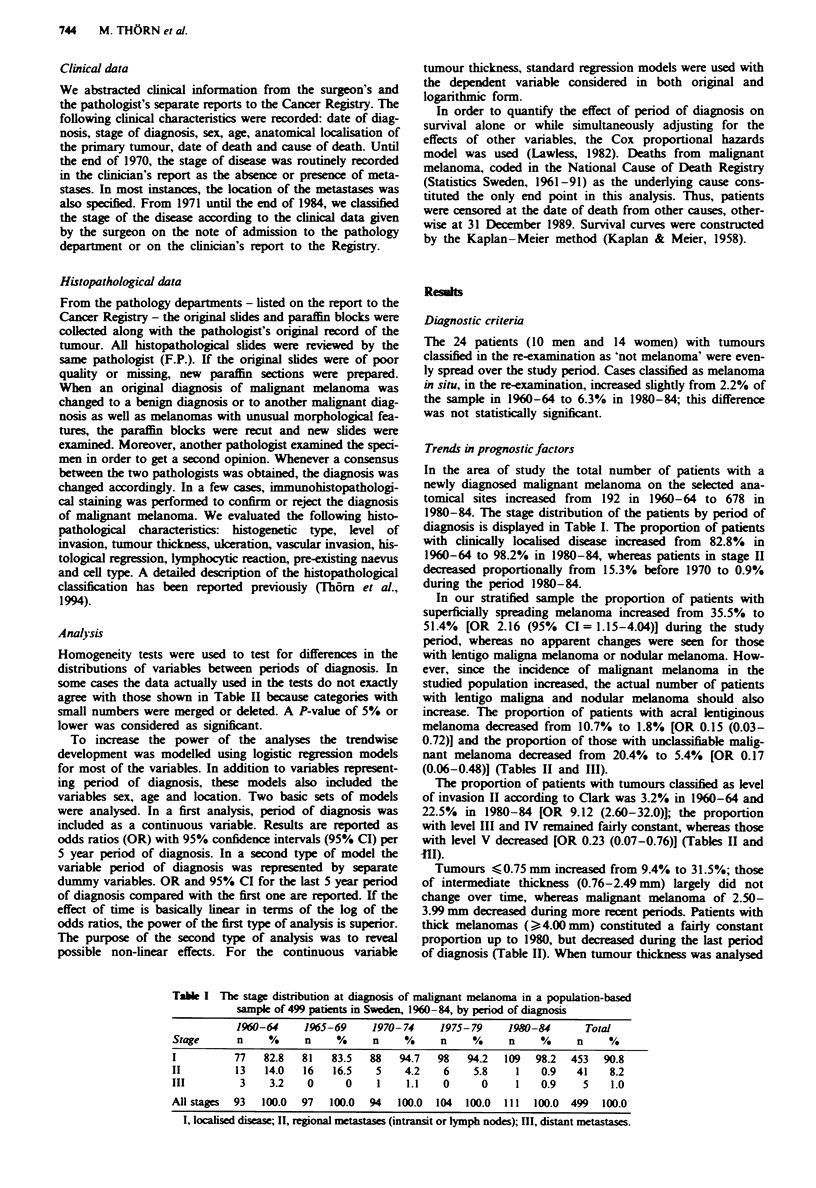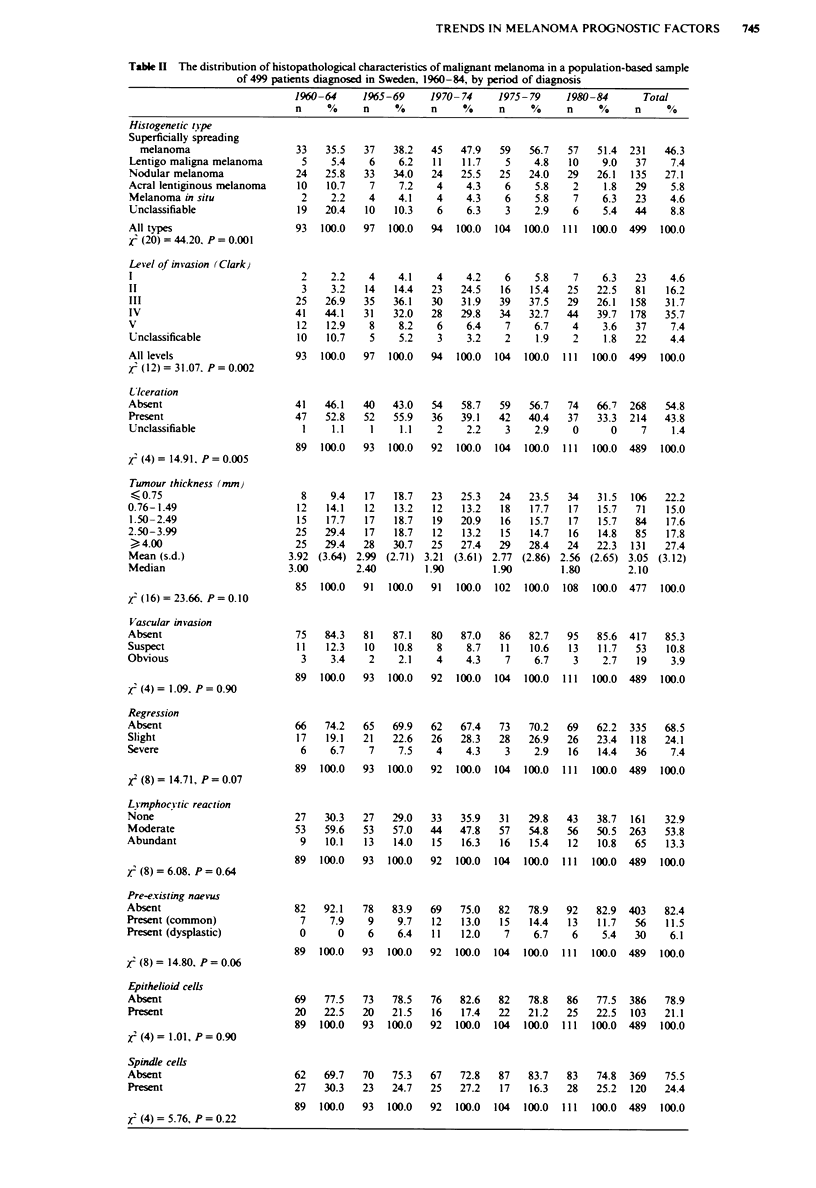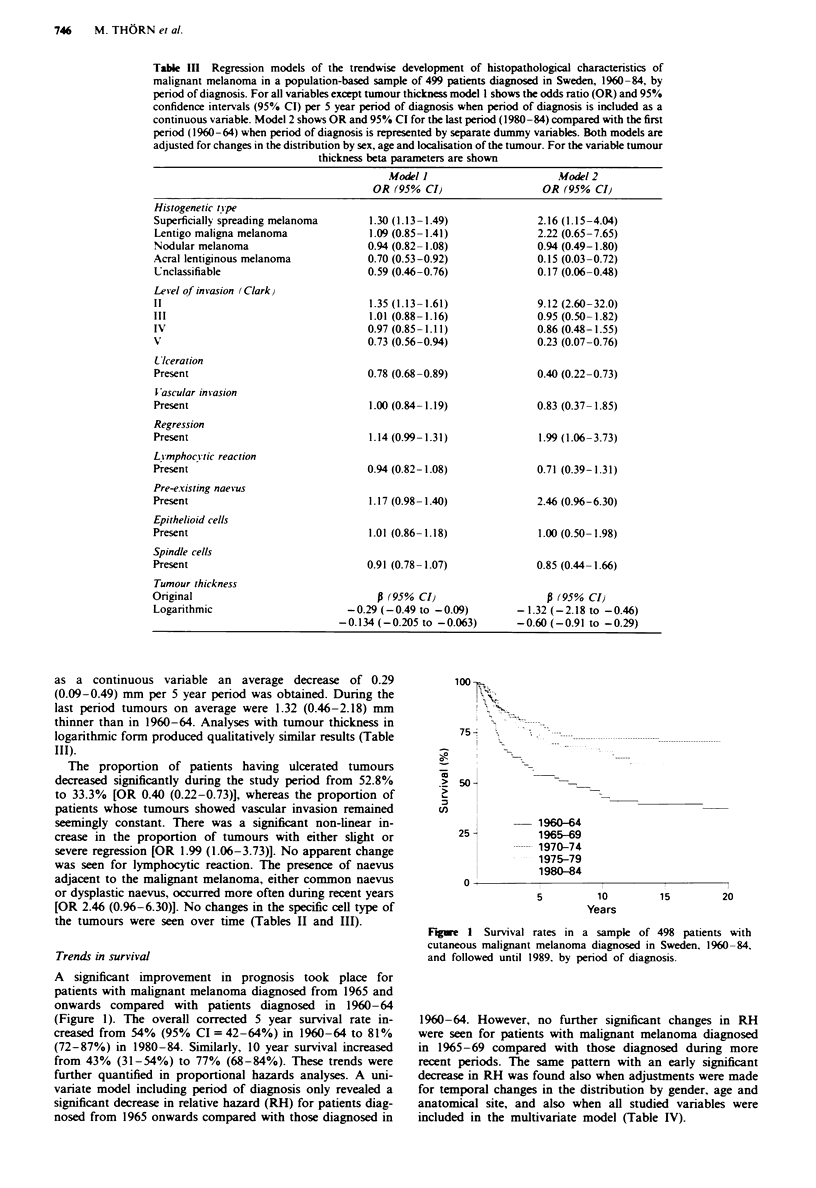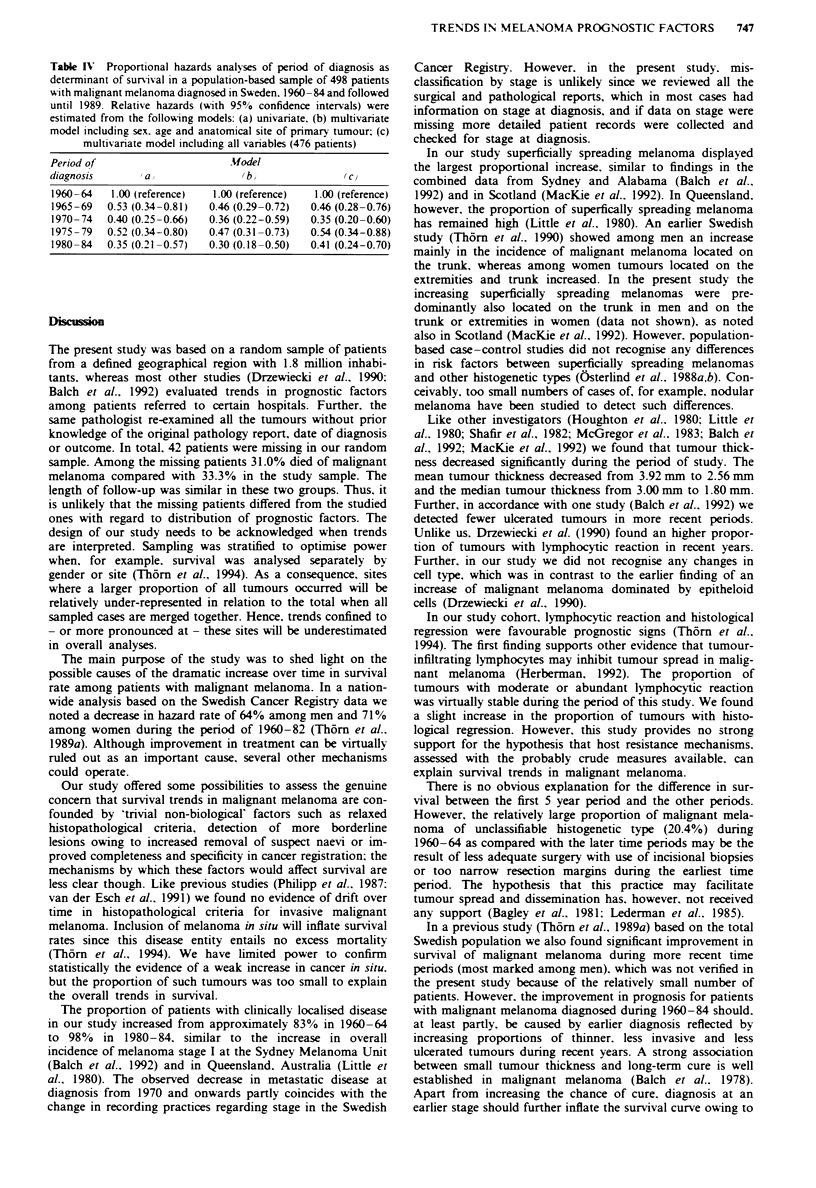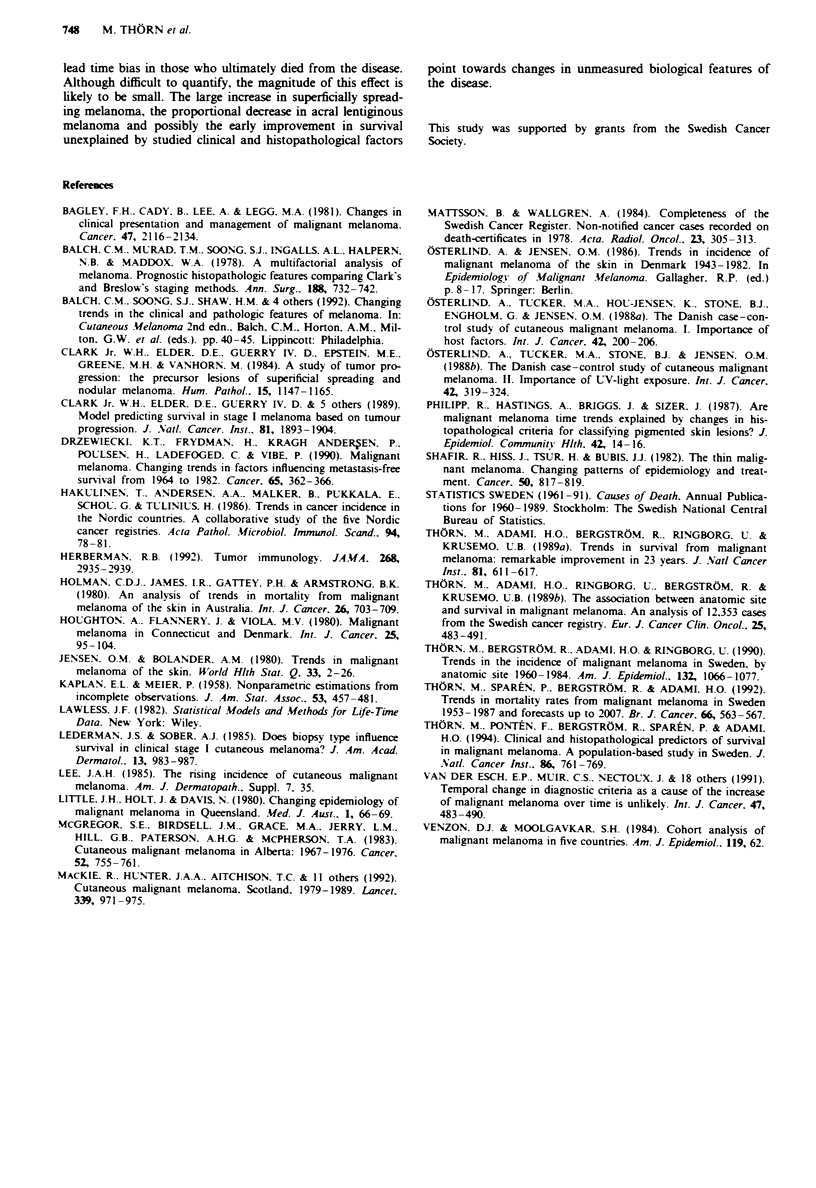# Trends in tumour characteristics and survival of malignant melanoma 1960-84: a population-based study in Sweden.

**DOI:** 10.1038/bjc.1994.388

**Published:** 1994-10

**Authors:** M. Thörn, F. Pontén, R. Bergström, P. Sparén, H. O. Adami

**Affiliations:** Department of Surgery, University Hospital, Uppsala, Sweden.

## Abstract

In Sweden, improvement in survival rates of patients with cutaneous malignant melanoma has counteracted the increase in incidence to produce a moderate rise in mortality. Our aim was to determine the possible impact of drift in diagnostic criteria, earlier diagnosis and changing biological features of the tumours upon trends in survival. We studied a stratified sample of 528 patients diagnosed between 1960 and 1984 in a strictly defined geographical region. No evidence of drift in diagnostic criteria was found. The proportion of patients with invasion level Clark II increased from 3.2% in 1960-64 to 22.5% in 1980-84, the proportion of thin melanomas (< or = 0.75 mm) increased from 9.4% to 31.5% and the tumour thickness decreased significantly between each 5 year period of diagnosis. These changes are most likely the results of earlier diagnosis. However, changes in tumour characteristics have occurred, since the proportion of superficially spreading malignant melanoma increased from 35% in 1960-64 to 51% in 1980-84 and the proportion of acral lentiginous melanoma decreased from 11% to 2%. The proportion of nodular melanomas remained fairly constant. The proportion of tumours with lymphocytic reaction did not change, whereas those with histological regression increased slightly. Proportional hazards analyses showed a significantly lower survival in patients diagnosed in 1960-64 but no apparent trend after 1965. This finding remained after adjustment for all studied clinical and histopathological factors which point towards changes in unmeasured biological features of the disease.


Br. J. Cancer (1994), 70, 743-748 © Macmillan Press Ltd., 1994

### Trends in tumour characteristics and survival of malignant melanoma 1960-84: a population-based study in Sweden

M. Thörn[1,2], F. Pontén[3], R. Bergström[2,4], P. Sparén[2] & H.-O. Adami[2,5]

[1]*Department of Surgery, University Hospital, S-751 85 Uppsala, Sweden;* [2]*Department of Cancer Epidemiology, University Hospital, S-751 85 Uppsala, Sweden;* [3]*Department of Pathology, University Hospital, S-751 85 Uppsala, Sweden;* [4]*Department of Statistics, Uppsala University, Box 513, S-751 20 Uppsala, Sweden;* [5]*Department of Epidemiology, Harvard School of Public Health, Boston, Massachusetts, USA.*

**Summary** In Sweden, improvement in survival rates of patients with cutaneous malignant melanoma has counteracted the increase in incidence to produce a moderate rise in mortality. Our aim was to determine the possible impact of drift in diagnostic criteria, earlier diagnosis and changing biological features of the tumours upon trends in survival. We studied a stratified sample of 528 patients diagnosed between 1960 and 1984 in a strictly defined geographical region. No evidence of drift in diagnostic criteria was found. The proportion of patients with invasion level Clark II increased from 3.2% in 1960-64 to 22.5% in 1980-84, the proportion of thin melanomas ($\leqslant$0.75 mm) increased from 9.4% to 31.5% and the tumour thickness decreased significantly between each 5 year period of diagnosis. These changes are most likely the results of earlier diagnosis. However, changes in tumour characteristics have occurred, since the proportion of superficially spreading malignant melanoma increased from 35% in 1960-64 to 51% in 1980-84 and the proportion of acral lentiginous melanoma decreased from 11% to 2%. The proportion of nodular melanomas remained fairly constant. The proportion of tumours with lymphocytic reaction did not change, whereas those with histological regression increased slightly. Proportional hazards analyses showed a significantly lower survival in patients diagnosed in 1960-64 but no apparent trend after 1965. This finding remained after adjustment for all studied clinical and histopathological factors which point towards changes in unmeasured biological features of the disease.

Many studies have reported increasing incidence rates of malignant melanoma during the last 30-40 years in countries with white populations (Jensen & Bolander, 1980; Lee, 1985; Hakulinen *et al.*, 1986; Østerlind *et al.*, 1986). These trends should be real since so far no apparent changes have been revealed in the histopathological criteria for the diagnosis of malignant melanoma (van der Esch *et al.*, 1991) and the mortality rates have also increased (Holman *et al.*, 1980; Venzon & Moolgavkar, 1984; Thörn *et al.*, 1992). However, the prognosis has improved in recent years, even after adjustment for temporal changes in distributions by sex, age and localisation of the primary tumour (Thörn *et al.*, 1989*a*).

The reasons for the rising survival rates are incompletely understood, especially since no therapeutic improvements have been established. Putative explanations are, firstly, increased public awareness of malignant melanoma, which may entail diagnosis of more tumours early when they are thin and curable. Secondly, and more speculatively, the biology and natural history of the disease might change over time, e.g. because other aetiological factors lead to a biologically less malignant phenotype. Studies on tumour progression in malignant melanoma suggest that precursor lesions play an important role and that the development of the invasive phenotype involves several steps (Clark *et al.*, 1984). Further, the metastatic capability may also differ between histologically invasive malignant melanomas (Clark *et al.*, 1989).

Our aim was to determine to what extent trends in survival are due mainly to drifts in diagnostic criteria, earlier diagnosis or changes in histopathological features such as histogenetic type or indicators of host defence mechanisms. We studied a sample from a population-based cohort of patients diagnosed between 1960 and 1984 with complete follow-up until 1989.

### Patients and methods

### *The cohort*

The Swedish Cancer Registry was created in 1958. All clinicians, pathologists and cytologists must report to the Registry any diagnosis of a malignant disease based on examination of surgically removed tissues or cytological specimens, biposies or autopsies. Nearly 100% of all cancers are diagnosed by one of these procedures, and in 95% of the cases both the clinician and the pathologist or cytologist notified the Registry (Mattson & Wallgren, 1984).

Our study was done in seven counties in central Sweden with approximately 1.8 million inhabitants. The goal was to draw a random sample of 12 patients from each of 50 subgroups defined by gender, five anatomical locations and five 5-year time periods of diagnosis from 1960 through 1984. The anatomical locations were defined as: eyelid and face; external ear and scalp-neck; trunk; upper extremity; lower extremity. The grouping of head-neck sites was based on earlier findings with similar prognoses in these site groups (Thörn *et al.*, 1989*b*). In total there were 2,093 eligible cases of malignant melanoma at the selected localisations. During the first 5 year period there were 192 eligible cases, during the second period 289 cases, during the third period 391 cases, during the fourth period 543 cases and during the fifth period 678 cases. Because the total number of patients in some subgroups was less than 12 during the earliest periods, there were 570 rather than 600 potentially eligible patients in the sample.

In 42 patients it was not possible to retrieve the original slides or paraffin blocks of the tumours. Thus, 528 (92.6%) of the sampled patients were analysed. Five of them had another cancer erroneously coded as cutaneous malignant melanoma. Further, 24 patients (ten men and 14 women) were excluded because they had tumours classified as 'not melanoma' in the histopathological review. These lesions consisted of various benign pigmented tumours and also malignant skin lesions other than melanoma. The trend analyses were based on 499 patients (247 men and 252 women). One patient was excluded from the proportional hazards analysis because of erroneously coded date of death. In ten patients (five men and five women) analysed separately the primary tumour was not known to the patient or to the doctor, most likely because of complete regression of the primary tumour. The multivariate analyses were based on 476 patients with complete information. All patients were followed until 31 December 1989, with respect to date and cause of death, by matching to the National Cause of Death Registry (Statistics Sweden, 1961-91).

Correspondence: M. Thörn.
Received 3 November 1993; and in revised form 10 April 1994.

*Clinical data*

We abstracted clinical information from the surgeon's and the pathologist's separate reports to the Cancer Registry. The following clinical characteristics were recorded: date of diagnosis, stage of diagnosis, sex, age, anatomical localisation of the primary tumour, date of death and cause of death. Until the end of 1970, the stage of disease was routinely recorded in the clinician's report as the absence or presence of metastases. In most instances, the location of the metastases was also specified. From 1971 until the end of 1984, we classified the stage of the disease according to the clinical data given by the surgeon on the note of admission to the pathology department or on the clinician's report to the Registry.

*Histopathological data*

From the pathology departments – listed on the report to the Cancer Registry – the original slides and paraffin blocks were collected along with the pathologist's original record of the tumour. All histopathological slides were reviewed by the same pathologist (F.P.). If the original slides were of poor quality or missing, new paraffin sections were prepared. When an original diagnosis of malignant melanoma was changed to a benign diagnosis or to another malignant diagnosis as well as melanomas with unusual morphological features, the paraffin blocks were recut and new slides were examined. Moreover, another pathologist examined the specimen in order to get a second opinion. Whenever a consensus between the two pathologists was obtained, the diagnosis was changed accordingly. In a few cases, immunohistopathological staining was performed to confirm or reject the diagnosis of malignant melanoma. We evaluated the following histopathological characteristics: histogenetic type, level of invasion, tumour thickness, ulceration, vascular invasion, histological regression, lymphocytic reaction, pre-existing naevus and cell type. A detailed description of the histopathological classification has been reported previously (Thörn et al., 1994).

*Analysis*

Homogeneity tests were used to test for differences in the distributions of variables between periods of diagnosis. In some cases the data actually used in the tests do not exactly agree with those shown in Table II because categories with small numbers were merged or deleted. A $P$-value of 5% or lower was considered as significant.

To increase the power of the analyses the trendwise development was modelled using logistic regression models for most of the variables. In addition to variables representing period of diagnosis, these models also included the variables sex, age and location. Two basic sets of models were analysed. In a first analysis, period of diagnosis was included as a continuous variable. Results are reported as odds ratios (OR) with 95% confidence intervals (95% CI) per 5 year period of diagnosis. In a second type of model the variable period of diagnosis was represented by separate dummy variables. OR and 95% CI for the last 5 year period of diagnosis compared with the first one are reported. If the effect of time is basically linear in terms of the log of the odds ratios, the power of the first type of analysis is superior. The purpose of the second type of analysis was to reveal possible non-linear effects. For the continuous variable tumour thickness, standard regression models were used with the dependent variable considered in both original and logarithmic form.

In order to quantify the effect of period of diagnosis on survival alone or while simultaneously adjusting for the effects of other variables, the Cox proportional hazards model was used (Lawless, 1982). Deaths from malignant melanoma, coded in the National Cause of Death Registry (Statistics Sweden, 1961–91) as the underlying cause constituted the only end point in this analysis. Thus, patients were censored at the date of death from other causes, otherwise at 31 December 1989. Survival curves were constructed by the Kaplan–Meier method (Kaplan & Meier, 1958).

### Results

*Diagnostic criteria*

The 24 patients (10 men and 14 women) with tumours classified in the re-examination as 'not melanoma' were evenly spread over the study period. Cases classified as melanoma *in situ*, in the re-examination, increased slightly from 2.2% of the sample in 1960–64 to 6.3% in 1980–84; this difference was not statistically significant.

*Trends in prognostic factors*

In the area of study the total number of patients with a newly diagnosed malignant melanoma on the selected anatomical sites increased from 192 in 1960–64 to 678 in 1980–84. The stage distribution of the patients by period of diagnosis is displayed in Table I. The proportion of patients with clinically localised disease increased from 82.8% in 1960–64 to 98.2% in 1980–84, whereas patients in stage II decreased proportionally from 15.3% before 1970 to 0.9% during the period 1980–84.

In our stratified sample the proportion of patients with superficially spreading melanoma increased from 35.5% to 51.4% [OR 2.16 (95% CI = 1.15–4.04)] during the study period, whereas no apparent changes were seen for those with lentigo maligna melanoma or nodular melanoma. However, since the incidence of malignant melanoma in the studied population increased, the actual number of patients with lentigo maligna and nodular melanoma should also increase. The proportion of patients with acral lentiginous melanoma decreased from 10.7% to 1.8% [OR 0.15 (0.03–0.72)] and the proportion of those with unclassifiable malignant melanoma decreased from 20.4% to 5.4% [OR 0.17 (0.06–0.48)] (Tables II and III).

The proportion of patients with tumours classified as level of invasion II according to Clark was 3.2% in 1960–64 and 22.5% in 1980–84 [OR 9.12 (2.60–32.0)]; the proportion with level III and IV remained fairly constant, whereas those with level V decreased [OR 0.23 (0.07–0.76)] (Tables II and III).

Tumours ≤0.75 mm increased from 9.4% to 31.5%; those of intermediate thickness (0.76–2.49 mm) largely did not change over time, whereas malignant melanoma of 2.50–3.99 mm decreased during more recent periods. Patients with thick melanomas (≥4.00 mm) constituted a fairly constant proportion up to 1980, but decreased during the last period of diagnosis (Table II). When tumour thickness was analysed

**Table I** The stage distribution at diagnosis of malignant melanoma in a population-based sample of 499 patients in Sweden, 1960–84, by period of diagnosis

| | *1960–64* | | *1965–69* | | *1970–74* | | *1975–79* | | *1980–84* | | *Total* | |
|---|---|---|---|---|---|---|---|---|---|---|---|---|
| *Stage* | n | % | n | % | n | % | n | % | n | % | n | % |
| I | 77 | 82.8 | 81 | 83.5 | 88 | 94.7 | 98 | 94.2 | 109 | 98.2 | 453 | 90.8 |
| II | 13 | 14.0 | 16 | 16.5 | 5 | 4.2 | 6 | 5.8 | 1 | 0.9 | 41 | 8.2 |
| III | 3 | 3.2 | 0 | 0 | 1 | 1.1 | 0 | 0 | 1 | 0.9 | 5 | 1.0 |
| All stages | 93 | 100.0 | 97 | 100.0 | 94 | 100.0 | 104 | 100.0 | 111 | 100.0 | 499 | 100.0 |

I, localised disease; II, regional metastases (intransit or lymph nodes); III, distant metastases.

**Table II** The distribution of histopathological characteristics of malignant melanoma in a population-based sample of 499 patients diagnosed in Sweden, 1960–84, by period of diagnosis

| | *1960–64* | | *1965–69* | | *1970–74* | | *1975–79* | | *1980–84* | | *Total* | |
|---|---|---|---|---|---|---|---|---|---|---|---|---|
| | n | % | n | % | n | % | n | % | n | % | n | % |
| *Histogenetic type* | | | | | | | | | | | | |
| Superficially spreading melanoma | 33 | 35.5 | 37 | 38.2 | 45 | 47.9 | 59 | 56.7 | 57 | 51.4 | 231 | 46.3 |
| Lentigo maligna melanoma | 5 | 5.4 | 6 | 6.2 | 11 | 11.7 | 5 | 4.8 | 10 | 9.0 | 37 | 7.4 |
| Nodular melanoma | 24 | 25.8 | 33 | 34.0 | 24 | 25.5 | 25 | 24.0 | 29 | 26.1 | 135 | 27.1 |
| Acral lentiginous melanoma | 10 | 10.7 | 7 | 7.2 | 4 | 4.3 | 6 | 5.8 | 2 | 1.8 | 29 | 5.8 |
| Melanoma *in situ* | 2 | 2.2 | 4 | 4.1 | 4 | 4.3 | 6 | 5.8 | 7 | 6.3 | 23 | 4.6 |
| Unclassifiable | 19 | 20.4 | 10 | 10.3 | 6 | 6.3 | 3 | 2.9 | 6 | 5.4 | 44 | 8.8 |
| All types | 93 | 100.0 | 97 | 100.0 | 94 | 100.0 | 104 | 100.0 | 111 | 100.0 | 499 | 100.0 |
| $\chi^2$ (20) = 44.20, $P = 0.001$ | | | | | | | | | | | | |
| *Level of invasion (Clark)* | | | | | | | | | | | | |
| I | 2 | 2.2 | 4 | 4.1 | 4 | 4.2 | 6 | 5.8 | 7 | 6.3 | 23 | 4.6 |
| II | 3 | 3.2 | 14 | 14.4 | 23 | 24.5 | 16 | 15.4 | 25 | 22.5 | 81 | 16.2 |
| III | 25 | 26.9 | 35 | 36.1 | 30 | 31.9 | 39 | 37.5 | 29 | 26.1 | 158 | 31.7 |
| IV | 41 | 44.1 | 31 | 32.0 | 28 | 29.8 | 34 | 32.7 | 44 | 39.7 | 178 | 35.7 |
| V | 12 | 12.9 | 8 | 8.2 | 6 | 6.4 | 7 | 6.7 | 4 | 3.6 | 37 | 7.4 |
| Unclassificable | 10 | 10.7 | 5 | 5.2 | 3 | 3.2 | 2 | 1.9 | 2 | 1.8 | 22 | 4.4 |
| All levels | 93 | 100.0 | 97 | 100.0 | 94 | 100.0 | 104 | 100.0 | 111 | 100.0 | 499 | 100.0 |
| $\chi^2$ (12) = 31.07, $P = 0.002$ | | | | | | | | | | | | |
| *Ulceration* | | | | | | | | | | | | |
| Absent | 41 | 46.1 | 40 | 43.0 | 54 | 58.7 | 59 | 56.7 | 74 | 66.7 | 268 | 54.8 |
| Present | 47 | 52.8 | 52 | 55.9 | 36 | 39.1 | 42 | 40.4 | 37 | 33.3 | 214 | 43.8 |
| Unclassifiable | 1 | 1.1 | 1 | 1.1 | 2 | 2.2 | 3 | 2.9 | 0 | 0 | 7 | 1.4 |
| | 89 | 100.0 | 93 | 100.0 | 92 | 100.0 | 104 | 100.0 | 111 | 100.0 | 489 | 100.0 |
| $\chi^2$ (4) = 14.91, $P = 0.005$ | | | | | | | | | | | | |
| *Tumour thickness (mm)* | | | | | | | | | | | | |
| ⩽0.75 | 8 | 9.4 | 17 | 18.7 | 23 | 25.3 | 24 | 23.5 | 34 | 31.5 | 106 | 22.2 |
| 0.76–1.49 | 12 | 14.1 | 12 | 13.2 | 12 | 13.2 | 18 | 17.7 | 17 | 15.7 | 71 | 15.0 |
| 1.50–2.49 | 15 | 17.7 | 17 | 18.7 | 19 | 20.9 | 16 | 15.7 | 17 | 15.7 | 84 | 17.6 |
| 2.50–3.99 | 25 | 29.4 | 17 | 18.7 | 12 | 13.2 | 15 | 14.7 | 16 | 14.8 | 85 | 17.8 |
| ⩾4.00 | 25 | 29.4 | 28 | 30.7 | 25 | 27.4 | 29 | 28.4 | 24 | 22.3 | 131 | 27.4 |
| Mean (s.d.) | 3.92 | (3.64) | 2.99 | (2.71) | 3.21 | (3.61) | 2.77 | (2.86) | 2.56 | (2.65) | 3.05 | (3.12) |
| Median | 3.00 | | 2.40 | | 1.90 | | 1.90 | | 1.80 | | 2.10 | |
| | 85 | 100.0 | 91 | 100.0 | 91 | 100.0 | 102 | 100.0 | 108 | 100.0 | 477 | 100.0 |
| $\chi^2$ (16) = 23.66, $P = 0.10$ | | | | | | | | | | | | |
| *Vascular invasion* | | | | | | | | | | | | |
| Absent | 75 | 84.3 | 81 | 87.1 | 80 | 87.0 | 86 | 82.7 | 95 | 85.6 | 417 | 85.3 |
| Suspect | 11 | 12.3 | 10 | 10.8 | 8 | 8.7 | 11 | 10.6 | 13 | 11.7 | 53 | 10.8 |
| Obvious | 3 | 3.4 | 2 | 2.1 | 4 | 4.3 | 7 | 6.7 | 3 | 2.7 | 19 | 3.9 |
| | 89 | 100.0 | 93 | 100.0 | 92 | 100.0 | 104 | 100.0 | 111 | 100.0 | 489 | 100.0 |
| $\chi^2$ (4) = 1.09, $P = 0.90$ | | | | | | | | | | | | |
| *Regression* | | | | | | | | | | | | |
| Absent | 66 | 74.2 | 65 | 69.9 | 62 | 67.4 | 73 | 70.2 | 69 | 62.2 | 335 | 68.5 |
| Slight | 17 | 19.1 | 21 | 22.6 | 26 | 28.3 | 28 | 26.9 | 26 | 23.4 | 118 | 24.1 |
| Severe | 6 | 6.7 | 7 | 7.5 | 4 | 4.3 | 3 | 2.9 | 16 | 14.4 | 36 | 7.4 |
| | 89 | 100.0 | 93 | 100.0 | 92 | 100.0 | 104 | 100.0 | 111 | 100.0 | 489 | 100.0 |
| $\chi^2$ (8) = 14.71, $P = 0.07$ | | | | | | | | | | | | |
| *Lymphocytic reaction* | | | | | | | | | | | | |
| None | 27 | 30.3 | 27 | 29.0 | 33 | 35.9 | 31 | 29.8 | 43 | 38.7 | 161 | 32.9 |
| Moderate | 53 | 59.6 | 53 | 57.0 | 44 | 47.8 | 57 | 54.8 | 56 | 50.5 | 263 | 53.8 |
| Abundant | 9 | 10.1 | 13 | 14.0 | 15 | 16.3 | 16 | 15.4 | 12 | 10.8 | 65 | 13.3 |
| | 89 | 100.0 | 93 | 100.0 | 92 | 100.0 | 104 | 100.0 | 111 | 100.0 | 489 | 100.0 |
| $\chi^2$ (8) = 6.08, $P = 0.64$ | | | | | | | | | | | | |
| *Pre-existing naevus* | | | | | | | | | | | | |
| Absent | 82 | 92.1 | 78 | 83.9 | 69 | 75.0 | 82 | 78.9 | 92 | 82.9 | 403 | 82.4 |
| Present (common) | 7 | 7.9 | 9 | 9.7 | 12 | 13.0 | 15 | 14.4 | 13 | 11.7 | 56 | 11.5 |
| Present (dysplastic) | 0 | 0 | 6 | 6.4 | 11 | 12.0 | 7 | 6.7 | 6 | 5.4 | 30 | 6.1 |
| | 89 | 100.0 | 93 | 100.0 | 92 | 100.0 | 104 | 100.0 | 111 | 100.0 | 489 | 100.0 |
| $\chi^2$ (8) = 14.80, $P = 0.06$ | | | | | | | | | | | | |
| *Epithelioid cells* | | | | | | | | | | | | |
| Absent | 69 | 77.5 | 73 | 78.5 | 76 | 82.6 | 82 | 78.8 | 86 | 77.5 | 386 | 78.9 |
| Present | 20 | 22.5 | 20 | 21.5 | 16 | 17.4 | 22 | 21.2 | 25 | 22.5 | 103 | 21.1 |
| | 89 | 100.0 | 93 | 100.0 | 92 | 100.0 | 104 | 100.0 | 111 | 100.0 | 489 | 100.0 |
| $\chi^2$ (4) = 1.01, $P = 0.90$ | | | | | | | | | | | | |
| *Spindle cells* | | | | | | | | | | | | |
| Absent | 62 | 69.7 | 70 | 75.3 | 67 | 72.8 | 87 | 83.7 | 83 | 74.8 | 369 | 75.5 |
| Present | 27 | 30.3 | 23 | 24.7 | 25 | 27.2 | 17 | 16.3 | 28 | 25.2 | 120 | 24.4 |
| | 89 | 100.0 | 93 | 100.0 | 92 | 100.0 | 104 | 100.0 | 111 | 100.0 | 489 | 100.0 |
| $\chi^2$ (4) = 5.76, $P = 0.22$ | | | | | | | | | | | | |

**Table III** Regression models of the trendwise development of histopathological characteristics of malignant melanoma in a population-based sample of 499 patients diagnosed in Sweden, 1960–84, by period of diagnosis. For all variables except tumour thickness model 1 shows the odds ratio (OR) and 95% confidence intervals (95% CI) per 5 year period of diagnosis when period of diagnosis is included as a continuous variable. Model 2 shows OR and 95% CI for the last period (1980–84) compared with the first period (1960–64) when period of diagnosis is represented by separate dummy variables. Both models are adjusted for changes in the distribution by sex, age and localisation of the tumour. For the variable tumour thickness beta parameters are shown

| | *Model 1* OR (95% CI) | *Model 2* OR (95% CI) |
|---|---|---|
| *Histogenetic type* | | |
| Superficially spreading melanoma | 1.30 (1.13–1.49) | 2.16 (1.15–4.04) |
| Lentigo maligna melanoma | 1.09 (0.85–1.41) | 2.22 (0.65–7.65) |
| Nodular melanoma | 0.94 (0.82–1.08) | 0.94 (0.49–1.80) |
| Acral lentiginous melanoma | 0.70 (0.53–0.92) | 0.15 (0.03–0.72) |
| Unclassifiable | 0.59 (0.46–0.76) | 0.17 (0.06–0.48) |
| *Level of invasion (Clark)* | | |
| II | 1.35 (1.13–1.61) | 9.12 (2.60–32.0) |
| III | 1.01 (0.88–1.16) | 0.95 (0.50–1.82) |
| IV | 0.97 (0.85–1.11) | 0.86 (0.48–1.55) |
| V | 0.73 (0.56–0.94) | 0.23 (0.07–0.76) |
| *Ulceration* | | |
| Present | 0.78 (0.68–0.89) | 0.40 (0.22–0.73) |
| *Vascular invasion* | | |
| Present | 1.00 (0.84–1.19) | 0.83 (0.37–1.85) |
| *Regression* | | |
| Present | 1.14 (0.99–1.31) | 1.99 (1.06–3.73) |
| *Lymphocytic reaction* | | |
| Present | 0.94 (0.82–1.08) | 0.71 (0.39–1.31) |
| *Pre-existing naevus* | | |
| Present | 1.17 (0.98–1.40) | 2.46 (0.96–6.30) |
| *Epithelioid cells* | | |
| Present | 1.01 (0.86–1.18) | 1.00 (0.50–1.98) |
| *Spindle cells* | | |
| Present | 0.91 (0.78–1.07) | 0.85 (0.44–1.66) |
| *Tumour thickness* | | |
| | *β (95% CI)* | *β (95% CI)* |
| Original | −0.29 (−0.49 to −0.09) | −1.32 (−2.18 to −0.46) |
| Logarithmic | −0.134 (−0.205 to −0.063) | −0.60 (−0.91 to −0.29) |

as a continuous variable an average decrease of 0.29 (0.09–0.49) mm per 5 year period was obtained. During the last period tumours on average were 1.32 (0.46–2.18) mm thinner than in 1960–64. Analyses with tumour thickness in logarithmic form produced qualitatively similar results (Table III).

The proportion of patients having ulcerated tumours decreased significantly during the study period from 52.8% to 33.3% [OR 0.40 (0.22–0.73)], whereas the proportion of patients whose tumours showed vascular invasion remained seemingly constant. There was a significant non-linear increase in the proportion of tumours with either slight or severe regression [OR 1.99 (1.06–3.73)]. No apparent change was seen for lymphocytic reaction. The presence of naevus adjacent to the malignant melanoma, either common naevus or dysplastic naevus, occurred more often during recent years [OR 2.46 (0.96–6.30)]. No changes in the specific cell type of the tumours were seen over time (Tables II and III).

*Trends in survival*

A significant improvement in prognosis took place for patients with malignant melanoma diagnosed from 1965 and onwards compared with patients diagnosed in 1960–64 (Figure 1). The overall corrected 5 year survival rate increased from 54% (95% CI = 42–64%) in 1960–64 to 81% (72–87%) in 1980–84. Similarly, 10 year survival increased from 43% (31–54%) to 77% (68–84%). These trends were further quantified in proportional hazards analyses. A univariate model including period of diagnosis only revealed a significant decrease in relative hazard (RH) for patients diagnosed from 1965 onwards compared with those diagnosed in

**Figure 1** Survival rates in a sample of 498 patients with cutaneous malignant melanoma diagnosed in Sweden, 1960–84, and followed until 1989, by period of diagnosis.

1960–64. However, no further significant changes in RH were seen for patients with malignant melanoma diagnosed in 1965–69 compared with those diagnosed during more recent periods. The same pattern with an early significant decrease in RH was found also when adjustments were made for temporal changes in the distribution by gender, age and anatomical site, and also when all studied variables were included in the multivariate model (Table IV).

**Table IV** Proportional hazards analyses of period of diagnosis as determinant of survival in a population-based sample of 498 patients with malignant melanoma diagnosed in Sweden, 1960–84 and followed until 1989. Relative hazards (with 95% confidence intervals) were estimated from the following models: (a) univariate, (b) multivariate model including sex, age and anatomical site of primary tumour; (c) multivariate model including all variables (476 patients)

| *Period of diagnosis* | *Model* (a) | (b) | (c) |
|---|---|---|---|
| 1960–64 | 1.00 (reference) | 1.00 (reference) | 1.00 (reference) |
| 1965–69 | 0.53 (0.34–0.81) | 0.46 (0.29–0.72) | 0.46 (0.28–0.76) |
| 1970–74 | 0.40 (0.25–0.66) | 0.36 (0.22–0.59) | 0.35 (0.20–0.60) |
| 1975–79 | 0.52 (0.34–0.80) | 0.47 (0.31–0.73) | 0.54 (0.34–0.88) |
| 1980–84 | 0.35 (0.21–0.57) | 0.30 (0.18–0.50) | 0.41 (0.24–0.70) |

### Discussion

The present study was based on a random sample of patients from a defined geographical region with 1.8 million inhabitants, whereas most other studies (Drzewiecki *et al.*, 1990; Balch *et al.*, 1992) evaluated trends in prognostic factors among patients referred to certain hospitals. Further, the same pathologist re-examined all the tumours without prior knowledge of the original pathology report, date of diagnosis or outcome. In total, 42 patients were missing in our random sample. Among the missing patients 31.0% died of malignant melanoma compared with 33.3% in the study sample. The length of follow-up was similar in these two groups. Thus, it is unlikely that the missing patients differed from the studied ones with regard to distribution of prognostic factors. The design of our study needs to be acknowledged when trends are interpreted. Sampling was stratified to optimise power when, for example, survival was analysed separately by gender or site (Thörn *et al.*, 1994). As a consequence, sites where a larger proportion of all tumours occurred will be relatively under-represented in relation to the total when all sampled cases are merged together. Hence, trends confined to – or more pronounced at – these sites will be underestimated in overall analyses.

The main purpose of the study was to shed light on the possible causes of the dramatic increase over time in survival rate among patients with malignant melanoma. In a nationwide analysis based on the Swedish Cancer Registry data we noted a decrease in hazard rate of 64% among men and 71% among women during the period of 1960–82 (Thörn *et al.*, 1989*a*). Although improvement in treatment can be virtually ruled out as an important cause, several other mechanisms could operate.

Our study offered some possibilities to assess the genuine concern that survival trends in malignant melanoma are confounded by 'trivial non-biological' factors such as relaxed histopathological criteria, detection of more borderline lesions owing to increased removal of suspect naevi or improved completeness and specificity in cancer registration; the mechanisms by which these factors would affect survival are less clear though. Like previous studies (Philipp *et al.*, 1987; van der Esch *et al.*, 1991) we found no evidence of drift over time in histopathological criteria for invasive malignant melanoma. Inclusion of melanoma *in situ* will inflate survival rates since this disease entity entails no excess mortality (Thörn *et al.*, 1994). We have limited power to confirm statistically the evidence of a weak increase in cancer *in situ*, but the proportion of such tumours was too small to explain the overall trends in survival.

The proportion of patients with clinically localised disease in our study increased from approximately 83% in 1960–64 to 98% in 1980–84, similar to the increase in overall incidence of melanoma stage I at the Sydney Melanoma Unit (Balch *et al.*, 1992) and in Queensland, Australia (Little *et al.*, 1980). The observed decrease in metastatic disease at diagnosis from 1970 and onwards partly coincides with the change in recording practices regarding stage in the Swedish Cancer Registry. However, in the present study, misclassification by stage is unlikely since we reviewed all the surgical and pathological reports, which in most cases had information on stage at diagnosis, and if data on stage were missing more detailed patient records were collected and checked for stage at diagnosis.

In our study superficially spreading melanoma displayed the largest proportional increase, similar to findings in the combined data from Sydney and Alabama (Balch *et al.*, 1992) and in Scotland (MacKie *et al.*, 1992). In Queensland, however, the proportion of superfically spreading melanoma has remained high (Little *et al.*, 1980). An earlier Swedish study (Thörn *et al.*, 1990) showed among men an increase mainly in the incidence of malignant melanoma located on the trunk, whereas among women tumours located on the extremities and trunk increased. In the present study the increasing superficially spreading melanomas were predominantly also located on the trunk in men and on the trunk or extremities in women (data not shown), as noted also in Scotland (MacKie *et al.*, 1992). However, population-based case–control studies did not recognise any differences in risk factors between superficially spreading melanomas and other histogenetic types (Österlind *et al.*, 1988*a*,*b*). Conceivably, too small numbers of cases of, for example, nodular melanoma have been studied to detect such differences.

Like other investigators (Houghton *et al.*, 1980; Little *et al.*, 1980; Shafir *et al.*, 1982; McGregor *et al.*, 1983; Balch *et al.*, 1992; MacKie *et al.*, 1992) we found that tumour thickness decreased significantly during the period of study. The mean tumour thickness decreased from 3.92 mm to 2.56 mm and the median tumour thickness from 3.00 mm to 1.80 mm. Further, in accordance with one study (Balch *et al.*, 1992) we detected fewer ulcerated tumours in more recent periods. Unlike us, Drzewiecki *et al.* (1990) found an higher proportion of tumours with lymphocytic reaction in recent years. Further, in our study we did not recognise any changes in cell type, which was in contrast to the earlier finding of an increase of malignant melanoma dominated by epitheloid cells (Drzewiecki *et al.*, 1990).

In our study cohort, lymphocytic reaction and histological regression were favourable prognostic signs (Thörn *et al.*, 1994). The first finding supports other evidence that tumour-infiltrating lymphocytes may inhibit tumour spread in malignant melanoma (Herberman, 1992). The proportion of tumours with moderate or abundant lymphocytic reaction was virtually stable during the period of this study. We found a slight increase in the proportion of tumours with histological regression. However, this study provides no strong support for the hypothesis that host resistance mechanisms, assessed with the probably crude measures available, can explain survival trends in malignant melanoma.

There is no obvious explanation for the difference in survival between the first 5 year period and the other periods. However, the relatively large proportion of malignant melanoma of unclassifiable histogenetic type (20.4%) during 1960–64 as compared with the later time periods may be the result of less adequate surgery with use of incisional biopsies or too narrow resection margins during the earliest time period. The hypothesis that this practice may facilitate tumour spread and dissemination has, however, not received any support (Bagley *et al.*, 1981; Lederman *et al.*, 1985).

In a previous study (Thörn *et al.*, 1989*a*) based on the total Swedish population we also found significant improvement in survival of malignant melanoma during more recent time periods (most marked among men), which was not verified in the present study because of the relatively small number of patients. However, the improvement in prognosis for patients with malignant melanoma diagnosed during 1960–84 should, at least partly, be caused by earlier diagnosis reflected by increasing proportions of thinner, less invasive and less ulcerated tumours during recent years. A strong association between small tumour thickness and long-term cure is well established in malignant melanoma (Balch *et al.*, 1978). Apart from increasing the chance of cure, diagnosis at an earlier stage should further inflate the survival curve owing to lead time bias in those who ultimately died from the disease. Although difficult to quantify, the magnitude of this effect is likely to be small. The large increase in superficially spreading melanoma, the proportional decrease in acral lentiginous melanoma and possibly the early improvement in survival unexplained by studied clinical and histopathological factors point towards changes in unmeasured biological features of the disease.

This study was supported by grants from the Swedish Cancer Society.

### References

BAGLEY, F.H., CADY, B., LEE, A. & LEGG, M.A. (1981). Changes in clinical presentation and management of malignant melanoma. *Cancer*, **47**, 2116–2134.

BALCH, C.M., MURAD, T.M., SOONG, S.J., INGALLS, A.L., HALPERN, N.B. & MADDOX, W.A. (1978). A multifactorial analysis of melanoma. Prognostic histopathologic features comparing Clark's and Breslow's staging methods. *Ann. Surg.*, **188**, 732–742.

BALCH, C.M., SOONG, S.J., SHAW, H.M. & 4 others (1992). Changing trends in the clinical and pathologic features of melanoma. In: *Cutaneous Melanoma* 2nd edn., Balch, C.M., Horton, A.M., Milton, G.W. *et al.* (eds.). pp. 40–45. Lippincott: Philadelphia.

CLARK Jr, W.H., ELDER, D.E., GUERRY IV, D., EPSTEIN, M.E., GREENE, M.H. & VANHORN, M. (1984). A study of tumor progression: the precursor lesions of superificial spreading and nodular melanoma. *Hum. Pathol.*, **15**, 1147–1165.

CLARK Jr, W.H., ELDER, D.E., GUERRY IV, D. & 5 others (1989). Model predicting survival in stage I melanoma based on tumour progression. *J. Natl. Cancer. Inst.*, **81**, 1893–1904.

DRZEWIECKI, K.T., FRYDMAN, H., KRAGH ANDERSEN, P., POULSEN, H., LADEFOGED, C. & VIBE, P. (1990). Malignant melanoma. Changing trends in factors influencing metastasis-free survival from 1964 to 1982. *Cancer*, **65**, 362–366.

HAKULINEN, T., ANDERSEN, A.A., MALKER, B., PUKKALA, E., SCHOU, G. & TULINIUS, H. (1986). Trends in cancer incidence in the Nordic countries. A collaborative study of the five Nordic cancer registries. *Acta Pathol. Microbiol. Immunol. Scand.*, **94**, 78–81.

HERBERMAN, R.B. (1992). Tumor immunology. *JAMA*, **268**, 2935–2939.

HOLMAN, C.D.J., JAMES, I.R., GATTEY, P.H. & ARMSTRONG, B.K. (1980). An analysis of trends in mortality from malignant melanoma of the skin in Australia. *Int. J. Cancer*, **26**, 703–709.

HOUGHTON, A., FLANNERY, J. & VIOLA, M.V. (1980). Malignant melanoma in Connecticut and Denmark. *Int. J. Cancer*, **25**, 95–104.

JENSEN, O.M. & BOLANDER, A.M. (1980). Trends in malignant melanoma of the skin. *World Hlth Stat. Q.*, **33**, 2–26.

KAPLAN, E.L. & MEIER, P. (1958). Nonparametric estimations from incomplete observations. *J. Am. Stat. Assoc.*, **53**, 457–481.

LAWLESS, J.F. (1982). *Statistical Models and Methods for Life-Time Data*. New York: Wiley.

LEDERMAN, J.S. & SOBER, A.J. (1985). Does biopsy type influence survival in clinical stage I cutaneous melanoma? *J. Am. Acad. Dermatol.*, **13**, 983–987.

LEE, J.A.H. (1985). The rising incidence of cutaneous malignant melanoma. *Am. J. Dermatopath.*, Suppl. 7, 35.

LITTLE, J.H., HOLT, J. & DAVIS, N. (1980). Changing epidemiology of malignant melanoma in Queensland. *Med. J. Aust.*, **1**, 66–69.

MCGREGOR, S.E., BIRDSELL, J.M., GRACE, M.A., JERRY, L.M., HILL, G.B., PATERSON, A.H.G. & MCPHERSON, T.A. (1983). Cutaneous malignant melanoma in Alberta: 1967–1976. *Cancer*, **52**, 755–761.

MACKIE, R., HUNTER, J.A.A., AITCHISON, T.C. & 11 others (1992). Cutaneous malignant melanoma, Scotland, 1979–1989. *Lancet*, **339**, 971–975.

MATTSSON, B. & WALLGREN, A. (1984). Completeness of the Swedish Cancer Register. Non-notified cancer cases recorded on death-certificates in 1978. *Acta. Radiol. Oncol.*, **23**, 305–313.

ÖSTERLIND, A. & JENSEN, O.M. (1986). Trends in incidence of malignant melanoma of the skin in Denmark 1943–1982. In *Epidemiology of Malignant Melanoma*. Gallagher, R.P. (ed.) p. 8–17. Springer: Berlin.

ÖSTERLIND, A., TUCKER, M.A., HOU-JENSEN, K., STONE, B.J., ENGHOLM, G. & JENSEN, O.M. (1988*a*). The Danish case–control study of cutaneous malignant melanoma. I. Importance of host factors. *Int. J. Cancer*, **42**, 200–206.

ÖSTERLIND, A., TUCKER, M.A., STONE, B.J. & JENSEN, O.M. (1988*b*). The Danish case–control study of cutaneous malignant melanoma. II. Importance of UV-light exposure. *Int. J. Cancer*, **42**, 319–324.

PHILIPP, R., HASTINGS, A., BRIGGS, J. & SIZER, J. (1987). Are malignant melanoma time trends explained by changes in histopathological criteria for classifying pigmented skin lesions? *J. Epidemiol. Community Hlth*, **42**, 14–16.

SHAFIR, R., HISS, J., TSUR, H. & BUBIS, J.J. (1982). The thin malignant melanoma. Changing patterns of epidemiology and treatment. *Cancer*, **50**, 817–819.

STATISTICS SWEDEN (1961–91). *Causes of Death*. Annual Publications for 1960–1989. Stockholm: The Swedish National Central Bureau of Statistics.

THÖRN, M., ADAMI, H.O., BERGSTRÖM, R., RINGBORG, U. & KRUSEMO, U.B. (1989*a*). Trends in survival from malignant melanoma: remarkable improvement in 23 years. *J. Natl Cancer Inst.*, **81**, 611–617.

THÖRN, M., ADAMI, H.O., RINGBORG, U., BERGSTRÖM, R. & KRUSEMO, U.B. (1989*b*). The association between anatomic site and survival in malignant melanoma. An analysis of 12,353 cases from the Swedish cancer registry. *Eur. J. Cancer Clin. Oncol.*, **25**, 483–491.

THÖRN, M., BERGSTRÖM, R., ADAMI, H.O. & RINGBORG, U. (1990). Trends in the incidence of malignant melanoma in Sweden, by anatomic site 1960–1984. *Am. J. Epidemiol.*, **132**, 1066–1077.

THÖRN, M., SPARÉN, P., BERGSTRÖM, R. & ADAMI, H.O. (1992). Trends in mortality rates from malignant melanoma in Sweden 1953–1987 and forecasts up to 2007. *Br. J. Cancer*, **66**, 563–567.

THÖRN, M., PONTÉN, F., BERGSTRÖM, R., SPARÉN, P. & ADAMI, H.O. (1994). Clinical and histopathological predictors of survival in malignant melanoma. A population-based study in Sweden. *J. Natl. Cancer Inst.*, **86**, 761–769.

VAN DER ESCH, E.P., MUIR, C.S., NECTOUX, J. & 18 others (1991). Temporal change in diagnostic criteria as a cause of the increase of malignant melanoma over time is unlikely. *Int. J. Cancer*, **47**, 483–490.

VENZON, D.J. & MOOLGAVKAR, S.H. (1984). Cohort analysis of malignant melanoma in five countries. *Am. J. Epidemiol.*, **119**, 62.